# FOXM1 promotes hepatocellular carcinoma progression by regulating KIF4A expression

**DOI:** 10.1186/s13046-019-1202-3

**Published:** 2019-05-09

**Authors:** Guohui Hu, Zhengwei Yan, Cheng Zhang, Minzhang Cheng, Yehong Yan, Yiting Wang, Libin Deng, Quqin Lu, Shiwen Luo

**Affiliations:** 1 Center for Experimental Medicine, the First Affiliated Hospital of Nanchang University, Nanchang, Jiangxi China; 2 Jiangxi Key Laboratory of Molecular Diagnostics and Precision Medicine, 17 Yongwai Street, Donghu District, Nanchang, 330006 Jiangxi China; 3 Department of General Surgery, the First Affiliated Hospital of Nanchang University, Nanchang, Jiangxi China; 4 Institute of Translational Medicine, Nanchang University, Nanchang, Jiangxi China; 5 Department of Epidemiology & Biostatistics, School of Public Health, Nanchang University, Nanchang, Jiangxi China

**Keywords:** FOXM1, KIF4A, Hepatocellular carcinoma, Proliferation

## Abstract

**Background:**

Forkhead box M1 (FOXM1) is a proliferation-associated transcription factor of the forkhead box proteins superfamily, which includes four isoforms FOXM1a, b, c, and d. FOXM1 has been implicated in hepatocellular carcinoma (HCC) progression, but the underlying molecular mechanism remains elusive. In this study, we aim to clarify the molecular basis for FOXM1-mediated HCC progression.

**Methods:**

Bioinformatic analysis was used to explore the differentially expressed genes predicting HCC proliferation. The expression of FOXM1 and kinesin family member (KIF)4A was confirmed by western blotting and immunohistochemistry in HCC tissues. Kaplan-Meier survival analysis was conducted to analyze the clinical impact of FOXM1 and KIF4A on HCC. The effect of FOXM1 on the regulation of KIF4A expression was studied in cell biology experiments. The interaction between KIF4A and FOXM1 was analyzed by chromatin immunoprecipitation and luciferase experiments. A series of experiments was performed to explore the functions of FOXM1/KIF4A in HCC progression, such as cell proliferation, cell growth, cell viability, and cell cycle. A xenograft mouse model was used to explore the regulatory effect of FOXM1-KIF4A axis on HCC tumor growth.

**Results:**

FOXM1 and KIF4A were overexpressed in human primary HCC tissues compared to that in matched adjacent normal liver tissue and are significant risk factors for HCC recurrence and shorter survival. We found that KIF4A was dominantly regulated by FOXM1c among the four isoforms, and further identified KIF4A as a direct downstream target of FOXM1c. Inhibiting FOXM1 decreased KIF4A expression in HCC cells, whereas its overexpression had the opposite effect. FOXM1-induced HCC cell proliferation was dependent on elevated KIF4A expression as KIF4A knockdown abolished FOXM1-induced proliferation of HCC cells both in vitro and in vivo.

**Conclusion:**

The FOXM1–KIF4A axis mediates human HCC progression and is a potential therapeutic target for HCC treatment.

**Electronic supplementary material:**

The online version of this article (10.1186/s13046-019-1202-3) contains supplementary material, which is available to authorized users.

## Background

Liver cancer is the fourth leading cause of cancer-related death worldwide [1]; hepatocellular carcinoma (HCC) is the most common type of liver cancer and has an extremely low 5-year overall survival rate. Most patients are diagnosed in advanced stages of the disease when treatment options are limited. There is, therefore, an urgent need to clarify the molecular mechanisms underlying HCC progression so that effective treatments can be developed.

Forkhead box M1 (FOXM1) is a typical proliferation-associated transcription factor belonging to the forkhead box superfamily of proteins. Four FOXM1 isoforms have been identified to date: FOXM1a acts as a repressor, FOXM1b and FOXM1c are transcriptional activators, while the cytoplasmic FOXM1d does not directly control transcription [2]. FOXM1 regulates the G1/S and G2/M transitions of the cell cycle [3]; its deletion during early embryonic development in mice was lethal between E14.5 and E16.5 due to failed liver and heart expansion, highlighting a role for FOXM1 in mitosis [4]. As a transcription factor, FOXM1 is regulated by the Hedgehog [5], p53 [6], Ras [7], and FoxO3 [8] signaling pathways and activates downstream targets including those that regulate the cell cycle and proliferation such as centromere protein (CENP) F, cyclin B1 (CCNB1), and kinesin family member (KIF)20A, among others [9]. FOXM1 has been identified in humans as a proto-oncogene [3, 10] whose overexpression is associated with the development of basal cell carcinoma under Hedgehog signaling [11]. FOXM1 and CENPF act synergistically to promote the malignant transformation of prostate cancer, and their co-expression is an indicator of poor survival and metastasis [12]. FOXM1 directly influences epithelial-mesenchymal transition (EMT) and pancreatic cancer cell invasion and metastasis [13] and contributes to paclitaxel resistance in breast cancer through regulation of its target gene KIF20A [14]. Various proteasome inhibitors (e.g., siomycin A and thiostrepton) targeting FOXM1 are used in cancer chemotherapy; however, they are associated with severe side effects [15].

KIFs are a highly conserved family of proteins present in all eukaryotes [16] that are classified as mitotic (involved in cell division) or non-mitotic (involved in intracellular transport) [17]. KIF4A plays a vital role in chromosome alignment and participates in anaphase of mitosis and cytokinesis [17]. Abnormal expression of KIF4A leads to aneuploidy [18]. KIF4A protein is highly expressed in fetal liver, spleen, thymus, and bone marrow, with lower levels detected in adult heart, small intestine, colon, kidney, and lung, as well as in the brain and other tissues [19]. KIF4A overexpression is observed in colorectal cancer [20] and pancreatic ductal adenocarcinoma [21] as well as in lung cancer, for which it is an independent prognostic risk factor [22]. KIF4A is also highly expressed in hepatitis B virus (HBV)-related liver cancer, and HBV stimulates KIF4A expression in HCC [23]. High levels of KIF4A are associated with poor prognosis in liver cancer [24] while KIF4A silencing suppressed the proliferation and migration of liver cancer cells [25], suggesting that KIF4A contributes to the malignant progression of liver cancer.

Based on the above findings and to clarify the molecular basis for liver cancer progression, the present study investigated the roles of FOXM1 and KIF4A and their relationship in HCC with in vitro experiments and by analyzing clinical specimens from patients. We identified KIF4A as a novel downstream target of FOXM1 and showed that the latter stimulates HCC cell proliferation by transcriptionally activating KIF4A. Our findings provide a basis for the development of more effective strategies for the treatment of HCC.

## Methods

### Antibodies, reagents, and constructs

Antibodies against the following proteins were used for western blotting: FOXM1 (Santa Cruz Biotechnology, CA, USA; sc-376471), KIF4A (Abcam, Cambridge, MA, USA; ab124903); CENPF (Abcam, Cambridge, MA, USA; ab90); CCNB1 (Cell Signaling Technology; Danvers, MA, USA; 4135); c-Myc (M4439) (Sigma-Aldrich, St. Louis, MO, USA); and glyceraldehyde 3-phosphate dehydrogenase (GAPDH) (Millipore, Billerica, MA, USA; MAB374). Normal goat anti-rabbit (31460) and goat anti-mouse (31430) IgG were purchased from Thermo Fisher Scientific (Waltham, MA, USA). Antibodies against KIF4A (sc-365145) and Ki67 (sc-15402) (both from Santa Cruz Biotechnology) were used for immunohistochemistry. An antibody against FOXM1 (Santa Cruz Biotechnology; sc-502) was used for both immunohistochemistry and Chromatin immunoprecipitation (ChIP) assay.

Puromycin (P8230) was purchased from Solarbio (Beijing, China). TRIzol reagent (15596018) and Lipofectamine 3000 transfection reagent (L3000–015) were from Thermo Fisher Scientific. Polyethylenimine transfection reagent (408727) was from Sigma-Aldrich (St. Louis, MO, USA). Other chemicals were of analytical grade and were from Sigma-Aldrich. A reverse transcription kit (RR047A) and real-time quantitative PCR assay kit (RR820A) (both from Takara Bio, Otsu, Japan) were used for mRNA quantitation. The Cell-light EdU (5-ethynyl-2′-deoxyuridine (EdU)) Apollo 567 In Vitro Kit (C10310–1) and Cell-light EdU Apollo 488 In Vitro Kit (C10310–3) were from RiboBio Biology (Guangzhou, China).

Expression plasmids for human different FOXM1 isoforms were subcloned into pcDNA3.1-A^+^ -myc/HisA mammalian expression vector using the In-Fusion Cloning kit (Clontech Laboratories, Mountain View, CA, USA; 639619) to generate pcDNA3.1-A^+^ -myc/HisA-FOXM1a, pcDNA3.1-A^+^ -myc/HisA-FOXM1b, pcDNA3.1-A^+^ -Myc/HisA-FOXM1c, and pcDNA3.1-A^+^ -myc/HisA-FOXM1d. FOXM1 and KIF4A short hairpin (sh)RNA constructs were generated with the sequences listed in Additional file 1: Table S2 using the BLOCK-iT Pol II miR-RNAi Expression Vector kit (Invitrogen, Carlsbad, CA, USA; K4936–00) according to the manufacturer’s instructions. Luciferase reporter constructs used to test the transcriptional activation of KIF4A by FOXM1 in the dual-luciferase assay were generated by inserting KIF4A promoter sequences containing FOXM1 binding sites into the pGL4.2-basic firefly luciferase vector (Promega, Madison, WI, USA; E6751). Cloned promoter sequences were validated by DNA sequencing. The primers used for the luciferase reporter constructs are listed in Additional file 1: Table S3.

### Bioinformatic analysis of gene expression data

For Bayesian analysis, clinical data of HCC samples including both tumor and non-tumor tissues were obtained from The Cancer Genome Atlas (TCGA) database (http://cancergenome.nih.gov/) and analyzed by using the makeContrasts function from the limma package (version 3.32.5) through R project (http://www.r-project.org). Genes with significant *p*-value and the top 50 were considered differentially expressed genes (DEGs).

For meta-analysis, the raw gene expression data of datasets (Additional file 1: Table S1) were downloaded from Gene Expression Omnibus (GEO) database (http://www.ncbi.nlm.nih.gov/geo/) based on the following inclusion criteria: a) dataset was specific to human HCC and included cancer and non-cancer tissues; b) more than 20 samples were involved in each group; c) all series had supplementary CEL data files available; d) all datasets were generated using Affymetrix U133 platform. The procedures used for meta-analysis were described as previously [26].

The prognostic value of DEGs in HCC was analyzed using the R project and R package (survival, stats, rms). Overall survival of the patient with high and low levels of DEGs was shown by using a Kaplan-Meier survival plot. The cut-off value of DEGs expression was determined by its mean RNA Seq v2 expression median value. The cBioPortal for Cancer Genomics data (http://www.cbioportal.org/) was used to analyze the correlation between FOXM1 and KIF4A expression levels.

### Cell lines and transfection

HEK293T cell and HepG2, Sk-hep1 human hepatocellular carcinoma cell lines were purchased from the American Type Culture Collection (Manassas, VA). Huh7 and Hep3B were obtained from the National Infrastructure of Cell Line Resource (Beijing, China). All cell lines were authenticated by short tandem repeat profiling and were found to be free of mycoplasma contamination. The cells were cultured in Dulbecco’s Modified Eagle’s Medium (HEK293T, Huh7, and HepG2) or Minimal Essential Medium (Hep3B and Sk-Hep1) (both from Gibco, Grand Island, NY, USA) supplemented with 10% fetal bovine serum (Gibco) and antibiotics (100 U/ml streptomycin and 100 μg/ml penicillin; Invitrogen) in a humidified incubator at 37 °C and 5% CO_2_. Cells were transiently transfected with Lipofectamine 3000 for HCC cell lines or with polyetherimide for HEK293T cells according to the manufacturer’s instructions. All stable HCC cell lines infected with lentivirus harboring FOXM1 or KIF4A overexpression or knockdown constructs were treated with 0.5–2 μg/ml puromycin dihydrochloride for 3 days, and selected clones were confirmed as positive by both PCR and western blotting.

### Western blotting and real-time PCR

Cells were harvested and lysed in radioimmunoprecipitation (RIPA) lysis buffer composed of 150 mM NaCl, 1% Nonidet P-40, 0.1% sodium dodecyl sulfate in phosphate-buffered saline (PBS), and protease inhibitor (pH 7.4). Cell lysates were cleared by centrifugation at 10,000×*g* for 10 min, and western blotting was performed. Total RNA was extracted using TRIzol reagent, and 1 μg was used to prepare cDNA by reverse transcription using PrimeScript RT reagent Kit with gDNA Eraser (Takara Bio; RR047A). Quantitative real-time PCR was carried out on an ABI StepOnePlus Real-Time PCR System (Applied Biosystems, Foster City, CA, USA) using SYBR Premix Ex Taq Tli RNaseH Plus (Takara Bio; RR820A) and the primers were shown in Additional file 1: Table S4. Data are presented as mean ± SD of at least three independent experiments.

### ChIP and luciferase assays

HepG2 cells grown to 90% confluence were cross-linked with 1% (v/v) formaldehyde. Chromatin was sonicated into fragments of 100 to 400 bp over six cycles of 10 s on /10 s off using a Bioruptor Sonicator (Diagenode, Denville, NJ, USA). The lysates were pre-cleared in bovine serum albumin-blocked protein A/G beads and incubated overnight with specific anti-FOXM1 antibody or control IgG. After washing, the DNA was eluted, and reverse cross-linked overnight at 65 °C. Eluted DNA was used as a template for semi-quantitative PCR. The input control was the supernatant before precipitation. The predicted binding sequences and primers used to amplify KIF4A promoter sequences are listed in Additional file 1: Table S5.

For the luciferase reporter assay, pGL4.2-basic-Luc reporter plasmids and the internal control plasmid pRL-TK were transfected into HepG2 cells grown to 70% confluence in 24-well plates. The FOXM1 expression plasmid or empty vector were co-transfected for 48 h, and reporter gene activity was assayed using the Dual Luciferase Assay System (Promega; E1910) according to the manufacturer’s instructions. The activity of the pGL4.2-basic-KIF4A promoter-luciferase reporter normalized to that of the pRL-TK Rluc reporter was compared between HepG2 cells transfected with FOXM1 expression plasmid or empty vector. The experiment was repeated three times.

### Cell proliferation, clonogenic, cell viability, and EdU-DNA synthesis assays

Cell proliferation rate was determined by a cell growth curve after counting cell numbers. Briefly, lentivirus-infected HCC cells were seeded in 24-well plates at a density of 2000 cells/well. The number of cells was quantified by flow cytometry every 24 h for 8 days, and the culture media was changed every 2 to 3 days. The results were shown as fold increase relative to the number of cells at day 1.

For the EdU-DNA synthesis assay, lentivirus-infected HCC cells were seeded in 96-well plates at a density of 8 × 10^3^ per well. After 24 h, the cell culture medium was replaced with 50 μM EdU solution diluted in the growth culture medium, followed by incubation for 2 h. The cells were then processed with the Cell-light EdU Apollo 567/488 In Vitro Kit according to the manufacturer’s instructions. Images were acquired on an inverted fluorescence microscope (IX71; Olympus, Tokyo, Japan) and analyzed with ImageJ software (National Institutes of Health, Bethesda, MD, USA). Experiments were performed in triplicate.

Cell viability was evaluated with the Cell Counting Kit (CCK)-8 assays (US Everbright, Suzhou, China; C6005) according to the manufacturer’s instructions. Briefly, lentivirus-infected HCC cells were seeded at a density of 1 × 10^3^ cells/well in 96-well culture plates. CCK-8 solution was added to each well at a final concentration of 10% and incubated for 2 h. Then the absorbance of the samples was measured at 450 nm using a Multiskan FC microplate reader (Thermo Fisher Scientific) every 24 h for 8 days. The culture media was changed every 2 to 3 days, and the results were shown with the value of OD_450_.

For the colony formation assay, lentivirus-infected HCC cells (3 × 10^3^ per well) were seeded in 6-well plates and cultured for about 2 weeks until the cells growing to visual colonies. The culture media was changed every 4 to 5 days during the cells’ growth time. The colonies were fixed and stained with 0.5% (w/v) crystal violet. The colonies were imaged with a scanner and quantified using ImageJ software.

### Flow cytometry and cell cycle analysis

For cell cycle analysis, cells infected with lentivirus were harvested and washed in PBS, and then fixed in ice-cold 70% ethanol at − 20 °C for 2 h. The cells were centrifuged at 1000 rpm for 3 min and washed with PBS, then resuspended in 0.5 ml PBS and treated with 100 μg/ml RNase A (Thermo Fisher Scientific; EN0531) and 50 μg/ml propidium iodide (Sigma-Aldrich; P4864) for 30 min at 4 °C in the dark. Cell cycle status was evaluated on an Accuri C6 Plus flow cytometer (BD Biosciences, San Jose, CA, USA), and data were analyzed using Modfit (Verity, Topsham, ME, USA) software programs.

### Lentivirus infection and xenograft mouse model

For lentivirus infection, Hep3B, Huh7, and HepG2 cells seeded at a density of 4 × 10^5^ were incubated with 1 × 10^8^ IU virus and 5 μg/ml polybrene (Sigma-Aldrich) for 24 h, then treated with 0.5-2 μg/ml puromycin for 72 h.

For in vivo experiments, 2 × 10^7^ Huh7 cells including Vector + sh-Control, FOXM1 + sh-Control, and FOXM1 + sh-KIF4A cell groups were injected into the flanks of 4-week-old female BALB/c-nu athymic nude mice (SLAC Laboratory Animal Co., Hunan, China; *n* = 8 mice per group). Subcutaneous tumor formation was observed starting 6 days post-injection, and tumor size was measured every 3 days using Vernier calipers. Tumor volume was calculated with the formula: (length × width^2^) / 2. At 24 days after injection, tumors were harvested for immunohistochemistry and western blotting. Protocols for animal experiments were approved by the Ethical Committee of the First Affiliated Hospital of Nanchang University and conformed to the guidelines of the National Institutes of Health on the ethical use of animals.

### Patients and clinical samples

Specimens and data from a cohort of 211 patients who underwent surgery for HCC cancer between January 2010 and June 2018 at the First Affiliated Hospital of Nanchang University were reviewed. All tumors were primary and untreated before surgery. Samples were reviewed by a pathologist to ensure that they included tumors and adjacent normal tissue. We defined tumor size as the maximum tumor diameter measured at the time of operation. The histologic type was defined according to the World Health Organization classification criteria as grade I (*n* = 17), grade II (*n* = 155), and grade III (*n* = 39). Clinical stage was defined according to the 7th edition of the American Joint Committee on Cancer. This work was approved by the Ethics Committee of the First Affiliated Hospital of Nanchang University (Nanchang, China).

### Immunohistochemistry and hematoxylin-eosin (HE) staining

Paraffin sections (3 μm) of formalin-fixed HCC and adjacent normal tissue mounted on glass slides were deparaffinized, rehydrated, and treated with 3% H_2_O_2_ for 10 min to block endogenous peroxidase activity. Antigen retrieval was performed by microwaving in EDTA buffer (pH 9.0) for 45 min followed by natural cooling. Tissue sections were incubated overnight at 4 °C with primary antibodies in a humidified chamber. The next morning, the slides were rinsed with PBS and then incubated for 60 min at 37 °C with the appropriate biotinylated secondary antibody (Zhongshan Biotechnology, Zhongshan, China), and immunoreactivity was visualized using the Polink-2 Horseradish Peroxidase DAB Detection kit (Zhongshan Biotechnology) according to the manufacturer’s protocol. Negative controls were prepared by replacing the primary antibody with normal IgG. For hematoxylin and eosin staining, the deparaffinized sides were nuclei stained with hematoxylin for 5 min, differentiate with 0.3% acid alcohol for 1 s, substituted with saturated lithium carbonate for 2 s, then followed by eosin staining. The Hematoxylin-Eosin Stain Solutions were from Yulu Experimental Company (Nanchang, China).

Immunohistochemical staining was evaluated by at least two independent investigators blinded to the histopathologic features of the samples. The German semi-quantitative scoring system was used to assess staining intensity and area. Each specimen was assigned a score according to the intensity of nuclear staining (no staining/not detected = 0; weak staining/light yellow = 1; moderate staining/yellowish brown = 2; and strong staining/brown =3) and the fraction of stained cells (0% = 0, 1–24% = 1, 25–49% = 2, 50–74% = 3, and 75–100% = 4). The final score was obtained by multiplying the two scores and ranged from 0 to 12. An FSX100 microscope equipped with a digital camera system (Olympus) was used to photograph the specimens.

### Statistical analysis

Differences in quantitative data between two groups for real-time PCR, Luciferase assay, in vitro assays including EdU assay, CCK-8, cell colony formation, and cell cycle analysis, and in vivo experiments of tumor weight were analyzed with Student t-test. The significance of the continuous cell growth curve and in vivo tumor volume between the groups was compared using one-way ANOVA analysis. The difference of the FOXM1 and KIF4A expression between tumor and adjacent normal tissues based on immunohistochemistry scores was determined by Wilcoxon matched-pairs signed-rank test. Immunohistochemistry scores between two or three independent groups were compared with the Mann–Whitney U test or Kruskal–Wallis H test. The χ^2^ test was used to analyze the correlation between gene expression and clinicopathological characteristics. The Kaplan–Meier method and log-rank test were used for survival analysis. *p* < 0.05 was considered statistically significant. All analyses were performed using SPSS v.22.0 software (SPSS Inc., Chicago, IL, USA).

## Results

### FOXM1 and KIF4A overexpression in human HCC is associated with poor outcome

**Fig. 1:**
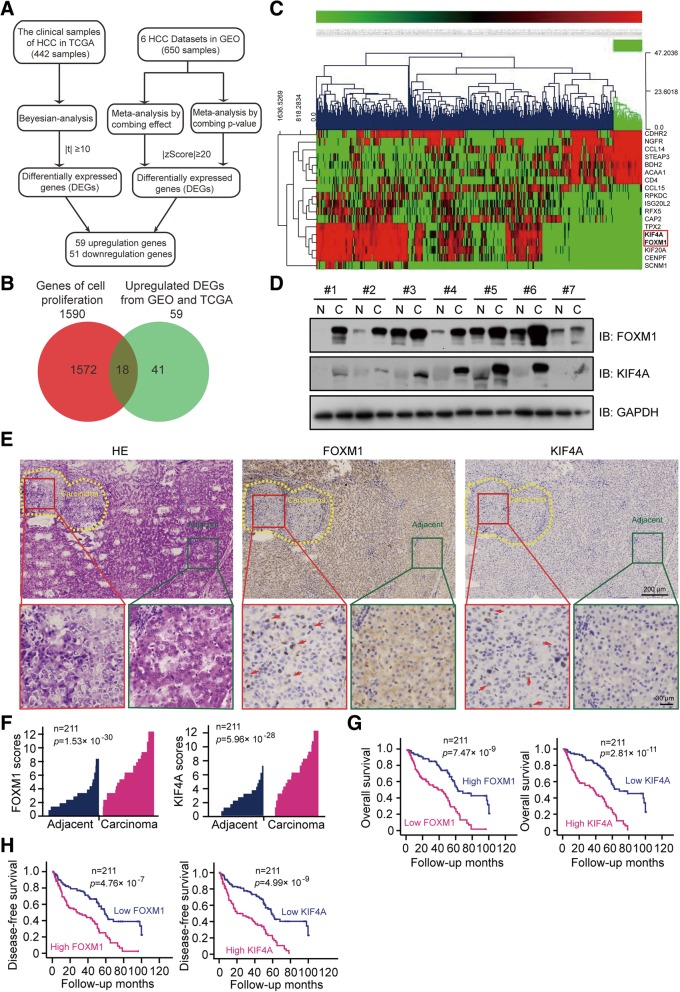
FOXM1 and KIF4A expression are elevated in HCC tissues. **a, b** Gene screening protocol for the bioinformatics analysis (**a**) and Venn diagram of differentially expressed genes (**b**) in the HCC cohort of TCGA and GEO datasets. **c** Heat map of genes extracted from the bioinformatics analysis (combined effects). **d** Western blotting analysis of FOXM1 and KIF4A expression in seven pairs of randomly selected HCC and matched adjacent non-tumor tissues. C, carcinoma tissue; N, matched adjacent non-tumor tissue. **e** HE staining and immunohistochemical detection of FOXM1 and KIF4A expression in a representative HCC and matched adjacent normal liver tissue samples. Subcellular locations of FOXM1 and KIF4A are indicated by red arrows. **f** FOXM1 and KIF4A expression plotted based on immunohistochemical score. Statistical significance was analyzed with the Wilcoxon matched-pairs signed-rank test. **g, h** Kaplan-Meier curves were used to evaluate differences between HCC patients with negative/low and medium/high FOXM1 and KIF4A expression in terms of overall survival (**g**) and disease-free survival (**h**)

We screened microarray datasets of HCC in the GEO (six datasets with a total of 650 samples) and HCC-related data from tumor and non-tumor tissues from TCGA (clinical samples of 422 patients). We evaluated TCGA and GEO data by Bayesian analysis and meta-analysis, respectively, and identified 110 differentially expressed genes, of which 59 were upregulated, and 51 were downregulated (Fig. 1a). Cluster analysis revealed that 18 of the upregulated genes including KIF4A, FOXM1, KIF20A, and CENPF are linked to HCC cell proliferation (Fig. 1b, c). A bioinformatics analysis of TCGA data confirmed the upregulation of FOXM1 and KIF4A (Additional file 2: Figure S1a, b) and the two FOXM1 target genes CENPF and KIF20A (Additional file 2: Figure S1c), and a correlation analysis of cBioPortal for Cancer Genomics data showed a positive correlation between FOXM1 and KIF4A expression levels (Additional file 2: Figure S1d). There was a similar correlation between FOXM1 and CENPF or KIF20A (Additional file 2: Figure S1e), suggesting that KIF4A maybe also act as a target of FOXM1, analogously to CENPF and KIF20A, in the FOXM1 regulatory network.

To investigate the roles of FOXM1 and KIF4A in HCC progression, we examined their protein levels in seven pairs of primary human HCC samples and matched adjacent normal liver tissues. The western blotting analysis revealed that FOXM1 and KIF4A protein expression was higher in HCC as compared to normal liver tissues (Fig. 1d), which was confirmed by immunohistochemistry (Fig. 1e, f), and corresponded to poor histological grade in HCC (Additional file 2: Figure S1f, g).

We investigated the correlation between FOXM1 and KIF4A expression and clinicopathological features of HCC by dividing the patients into high and low expression groups according to mean immunohistochemistry scores. High expression of FOXM1 was positively correlated with poor histological grade and advanced tumor–node–metastasis (TNM) stage in HCC; while elevated KIF4A level was positively correlated with vascular invasion, as well as poor histological grade and late TNM stage (Table 1). To determine the significance of FOXM1 and KIF4A overexpression concerning HCC patient prognosis, we analyzed overall survival and disease-free survival by the Kaplan–Meier method and log-rank test and found that patients with higher levels of these two proteins had lower survival rates (Fig. 1g, h). Consistent with these results, an analysis of 369 HCC patient samples from TCGA database showed that upregulation of FOXM1 or KIF4A was associated with shorter survival (Additional file 2: Figure S1 h, i). Thus, FOXM1 and KIF4A are co-expressed in HCC tissue and may be independent predictive and prognostic biomarkers for HCC.

**Table 1:** Association of FOXM1 and KIF4A expression levels with different clinicopathologic characteristics in HCC

Clinicopathologic	FOXM1 expression			KIF4A expression		
	Low	High	*p* value	Low	High	*p* value
	Count (n%)	Count (n%)		Count (n%)	Count (n%)	
Sex						
Male	89 (82.4)	89 (86.4)	0.424	92 (80.7)	86 (88.7)	0.113
Female	19 (17.6)	14 (13.6)		22 (19.3)	11 (11.3)	
Age, years						
≤ 50	53 (49.1)	50 (48.5)	0.939	53 (46.5)	50 (51.5)	0.464
>50	55 (50.9)	53 (51.5)		61 (53.5)	47 (48.5)	
AFP Level, ug/L						
≤ 20	35 (32.4)	26 (25.2)	0.393	39 (34.2)	22 (22.7)	0.148
>20	67 (62.0)	73 (70.9)		69 (60.5)	71 (73.2)	
untested	6 (5.6)	4 (3.9)		6 (5.3)	4 (4.1)	
CA-199, U/mL						
≤ 27	69 (63.9)	69 (67.0)	0.177	72 (63.2)	66 (68.0)	0.393
>27	17 (15.7)	22 (21.3)		20 (17.5)	19 (19.6)	
untested	22 (20.4)	12 (11.7)		22 (19.3)	12 (12.4)	
ALB, g/L						
<34	90 (83.3)	87 (84.5)	0.823	96 (84.2)	81 (83.5)	0.890
≥ 34	18 (16.7)	16 (15.5)		18 (15.8)	16 (16.5)	
Pre-S1						
Negative	17 (15.7)	11 (10.7)	0.446	17 (14.9)	11 (11.3)	0.516
Positive	38 (35.2)	34 (33.0)		41 (36.0)	31 (32.0)	
Unknown	53 (49.1)	58 (56.3)		56 (49.1)	55 (56.7)	
TBIL, umol/L						
≤ 20.5	98 (90.7)	90 (87.4)	0.433	104 (91.2)	84 (85.6)	0.282
>20.5	10 (9.3)	13 (12.6)		10 (8.8)	13 (13.4)	
Cirrhosis						
No	42 (38.9)	50 (48.5)	0.157	46 (40.4)	46 (47.4)	0.302
Yes	66 (61.1)	53 (51.5)		68 (59.6)	51 (52.6)	
HBV infection						
Negative	11 (10.2)	8 (7.8)	0.540	11 (9.6)	8 (8.2)	0.723
Positive	97 (89.8)	95 (92.2)		103 (90.4)	89 (91.8)	
Histologic grade						
Well	14 (13.0)	3 (2.9)	**0.003**	15 (13.1)	2 (2.0)	**0.001**
Moderate	81 (75.0)	74 (71.9)		85 (74.6)	70 (72.2)	
Poor	13 (12.0)	26 (25.2)		14 (12.3)	25 (25.8)	
Vascular invasion						
Negative	89 (82.4)	86 (83.5)	0.834	100 (87.7)	75 (77.3)	**0.045**
Positive	19 (17.6)	17 (16.5)		14 (12.3)	22 (22.7)	
TNM stage						
StageI-II	65 (60.2)	31 (30.1)	**1.15 × 10** ^**–5**^	70 (61.4)	26 (26.8)	**4.91 × 10** ^**–7**^
StageIII-IV	43 (39.8)	72 (69.9)		44 (38.6)	71 (73.2)	

*AFP* alpha fetoprotein, *ALB* serum albumin, *TBIL* total bilirubin, *HBV* hepatitis B virus, *TNM* tumor-node-metastasis

*p* values were calculated by comparing the expression of FOXM1 and KIF4A with different clinical variables respectively using a chi-square test. *p* < 0.05 was considered statistically significant

### FOXM1 positively regulates KIF4A expression

**Fig. 2:**
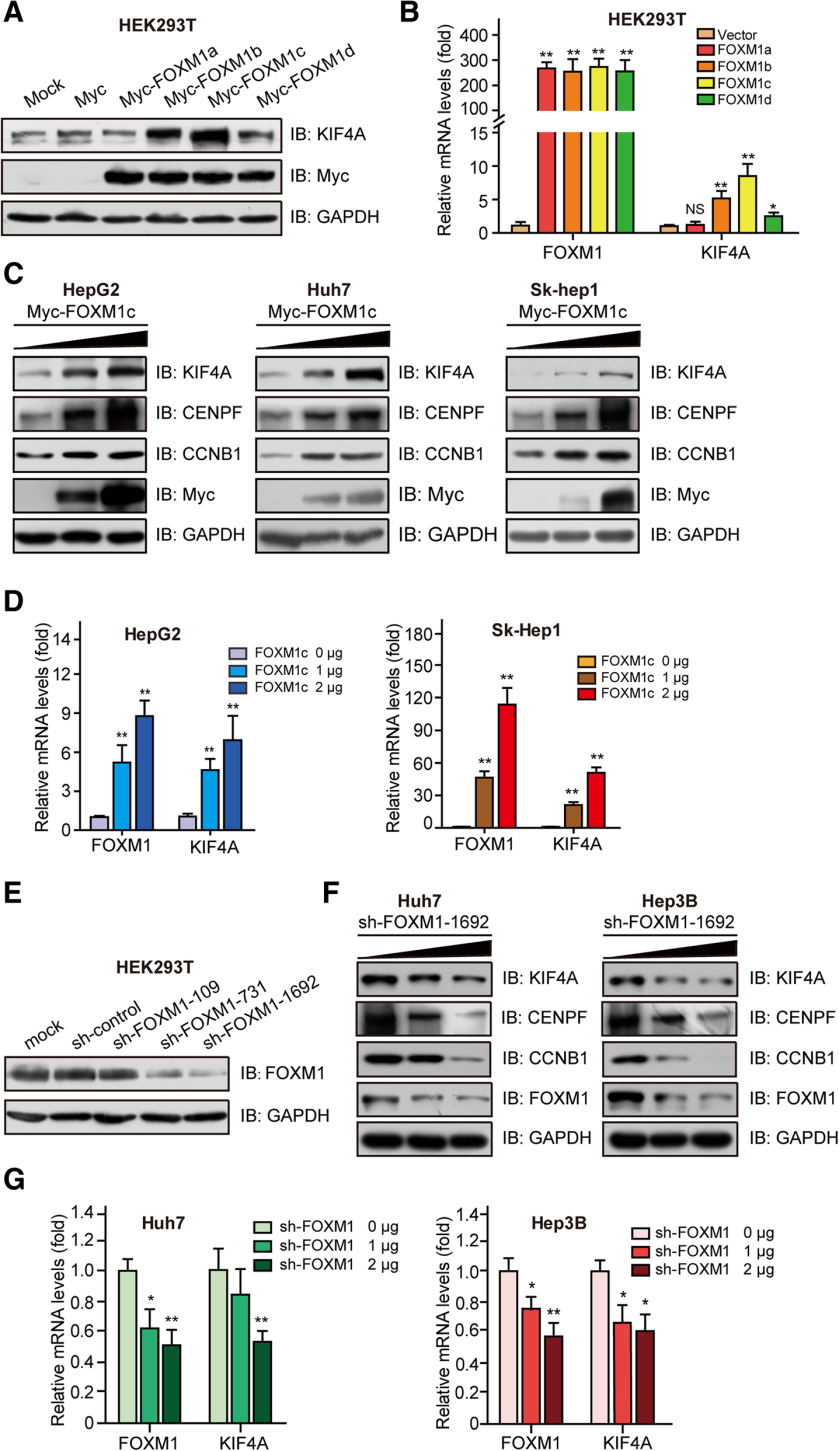
FOXM1 regulates KIF4A expression. **a**, **b** HEK293T cells were transfected with overexpression plasmids for specific isoforms of FOXM1 for 48 h and then harvested for western blotting with indicated antibodies (**a**) and quantitative real-time PCR (**b**). **c, d** HepG2, Huh7, and Sk-Hep1 cells were transfected with pcDNA 3.1-Myc/HisA-vector or pcDNA 3.1-Myc/HisA-FOXM1c plasmids for 48 h and then harvested for western blotting with indicated antibodies (**c**) and quantitative real-time PCR (**d**). **e** HEK293T cells transfected with shRNA-Control or shRNA-FOXM1 constructs for 48 h were harvested for western blotting with indicated antibodies. **f, g** Huh7, and Hep3B cells were transfected with sh-Control or sh-FOXM1 for 48 h and then harvested for western blotting (**f**) with indicated antibodies and quantitative real-time PCR (**g**). CENPF and CCNB1 were used as a positive control. Data are shown as mean ± SD (*n* = 3). *p < 0.05, **p < 0.01

We generated FOXM1 overexpression constructs targeting the different isoforms and transfected these into HEK293T cells. We found that overexpression of the transcriptional repressor FOXM1a did not induce the expression of KIF4A; FOXM1d induced KIF4A expression a little, while FOXM1b and FOXM1c markedly increased KIF4A mRNA and protein expression, with FOXM1c exerting the most potent effect (Fig. 2a, b). Thus, we focused on FOXM1c (hereafter referred to as FOXM1) as it was previously reported to be highly expressed in pancreatic tumors [27] and to promote EMT and metastasis [28]. To determine whether FOXM1 regulates KIF4A expression in HCC, we transfected different HCC cell lines with the FOXM1 overexpression construct, which increased KIF4A mRNA and protein levels (Fig. 2c, d). Conversely, shRNA-mediated knockdown of FOXM1 had the opposite effect (Fig. 2e–g), indicating that FOXM1 regulates KIF4A expression.

### FOXM1 binds to the KIF4A promoter

**Fig. 3:**
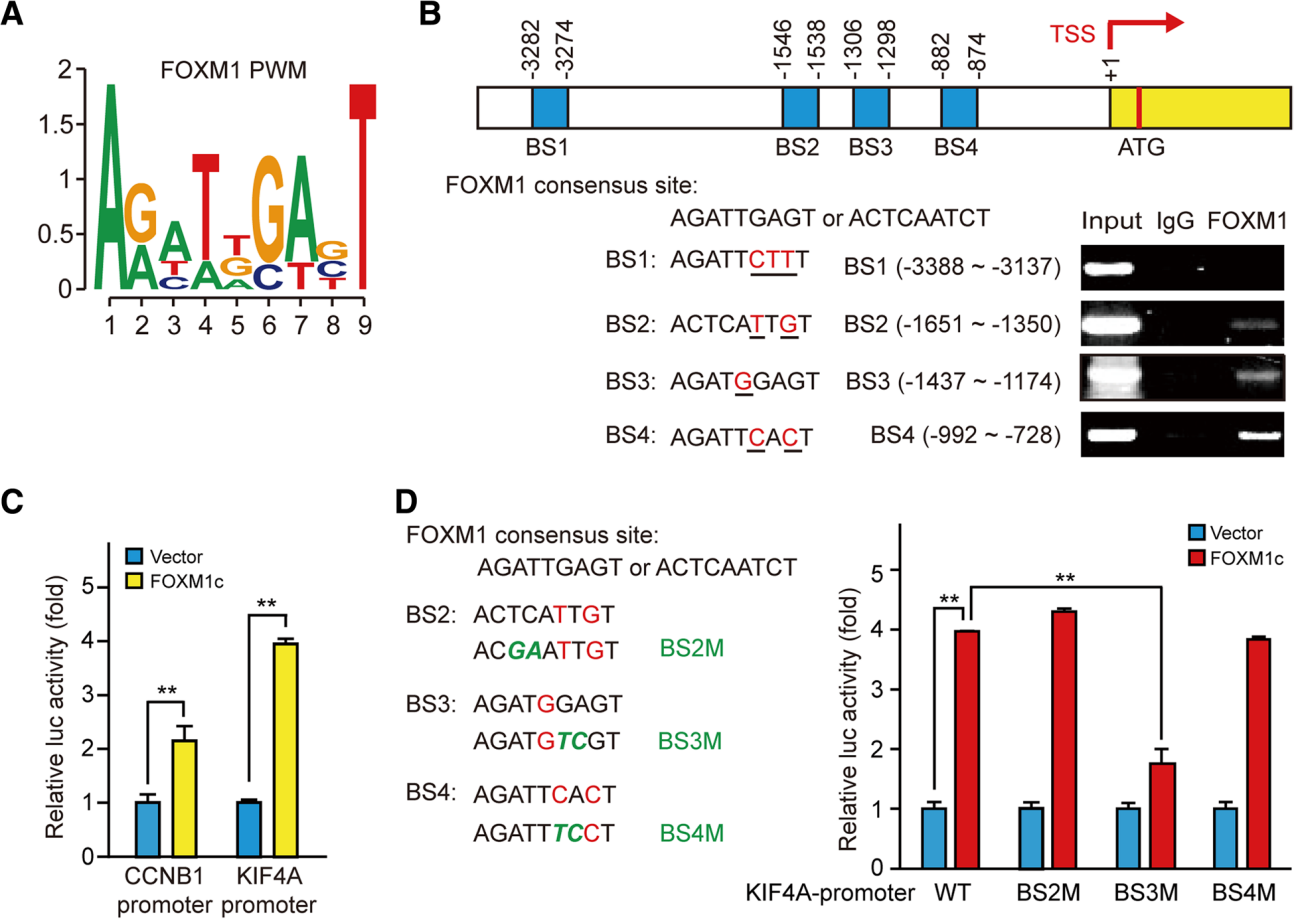
FOXM1 directly binds to the KIF4A promoter and activates gene transcription. **a** Map of FOXM1 binding site sequence. **b** Schematic illustration of four potential FOXM1 binding sites (BS1, BS2, BS3, and BS4) in the KIF4A promoter (top). The 9-base pair sequence of the FOXM1 consensus site and sequences of four FOXM1-binding sites in the KIF4A promoter are shown. Chromatin was isolated from HepG2 cells, and ChIP assay was performed with goat IgG control and FOXM1-specific antibodies (bottom). **c** Full-length KIF4A luciferase construct was transfected into HepG2 cells with empty vector or the FOXM1 overexpression plasmid for 48 h. Transcriptional activation was analyzed with the dual luciferase reporter assay, with human CCNB1-luciferase construct used as a positive control. **d** Luciferase constructs for wild-type, or mutant KIF4A promoter was transfected into HepG2 cells along with FOXM1 overexpression plasmid or control vector for 48 h. Transcriptional activation was analyzed with the dual luciferase reporter assay. Results were normalized to Renilla luciferase activity and are expressed as fold change in luciferase activity relative to the control. Data represent the mean ± SD of three independent experiments. **p < 0.01

To confirm KIF4A as a target gene of FOXM1, we used the MatInspector program of Genomatix (http://www.genomatix.de/) [29] to predict FOXM1 binding site(s) in the KIF4A promoter. We confirmed three of the four predicted sites (BS2, − 1651 to − 1350; BS3, − 1437 to − 1174; and BS4, − 992 to − 728) by ChIP assay (Fig. 3a, b). We next constructed a set of luciferase reporter vectors containing full-length or mutated KIF4A promoters to evaluate direct FOXM1 binding. In FOXM1-overexpressing cells, the full-length promoter construct showed increased luciferase activity that was comparable to that of the positive control CCNB1, a known FOXM1 target gene [30] (Fig. 3c), indicating that FOXM1 directly activates *KIF4A* gene transcription. Accordingly, mutation of BS3 abolished the luciferase activity induced by FOXM1, whereas mutation of the other two sites did not affect (Fig. 3d). These results indicate that KIF4A is a direct transcriptional target of FOXM1, which binds to the BS3 site of the KIF4A promoter.

### FOXM1 and KIF4A promote human HCC cell proliferation

**Fig. 4:**
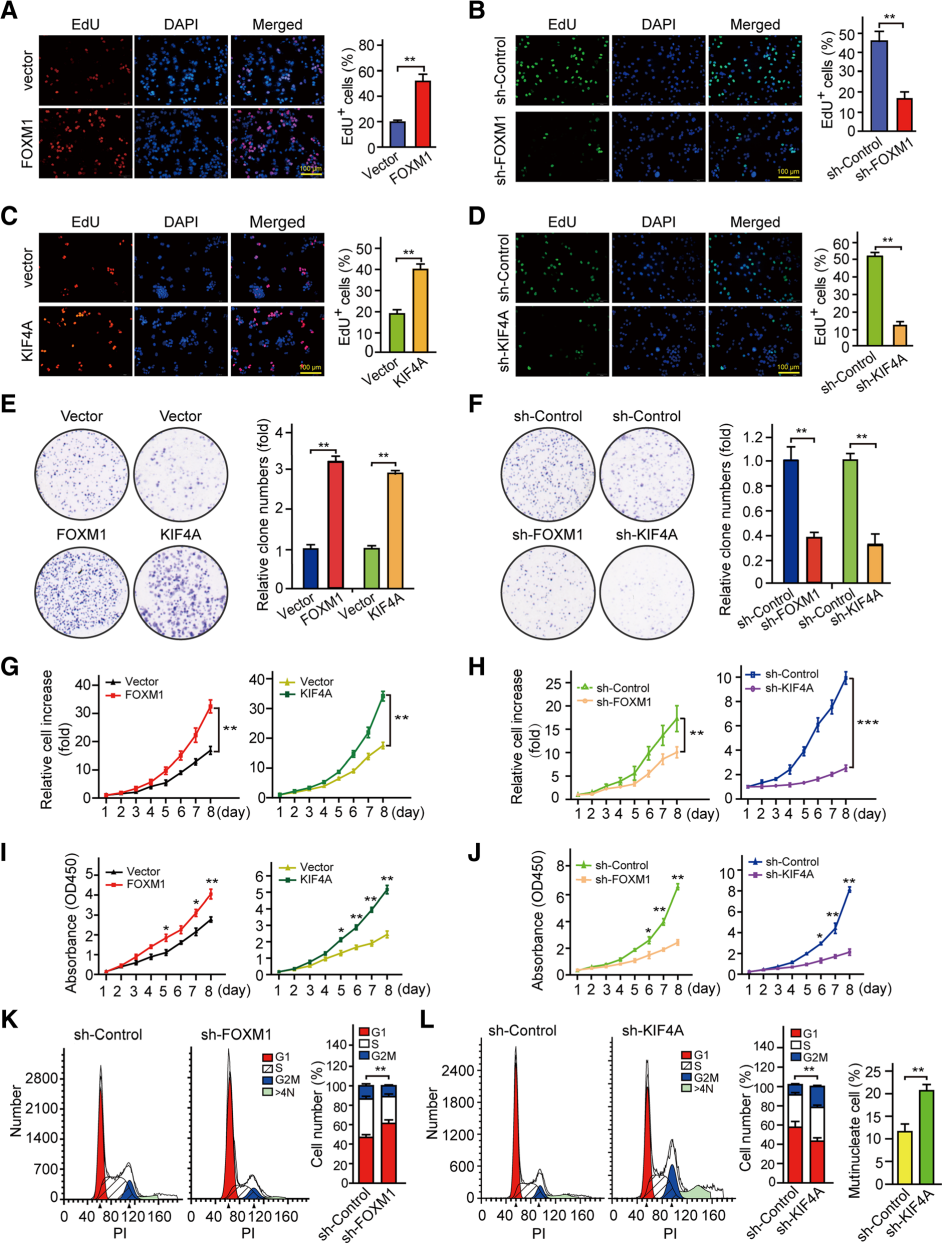
FOXM1 and KIF4A promote HCC cell proliferation. **a, b** FOXM1 overexpression increases the proliferation rate of HepG2 cells (**a**) whereas FOXM1 knockdown with specific shRNA decreases the proliferation rate of Huh7 cells (**b**), as shown by EdU staining. **c, d** KIF4A overexpression increases the proliferation rate of HepG2 cells (**c**) whereas KIF4A knockdown (sh-KIF4A) decreases the proliferation rate of Huh7 cells (**d**), as shown by EdU staining. **e, f** FOXM1 or KIF4A overexpression increases colony formation by HepG2 cells (**e**) whereas their knockdown decreases colony formation by Huh7 cells (**f**). **g, h** FOXM1 or KIF4A overexpression increases the cell growth rate of HepG2 cells (**g**) whereas their knockdown decreases (**h**) the cell growth rate of Huh7 cells, as indicated by fold change relative to the initial time point. **i, j** Increased the viability of HepG2 cells upon FOXM1 or KIF4A overexpression and decreased the viability of Huh7 cells upon FOXM1 or KIF4A knockdown, as determined with CCK-8. **k** Cell cycle profile of FOXM1-knockdown Hep3B cells determined by flow cytometry analysis with propidium iodide (PI) staining (left); the proportion of cells in each phase of the cell cycle was quantified concerning the total number of cells (right). **l** Flow cytometry analysis was used to determine the effect of KIF4A knockdown on cell cycle distribution (left) in Huh7 cells; the fraction of cells in each phase (middle) and the number of multinucleate cells (right) are shown as a histogram. Data represent the mean ± SD of three independent experiments. *p < 0.05, **p < 0.01

Given that FOXM1 and KIF4A are factors that potentially affect HCC cell proliferation (Fig. 1a, b) and based on reports that FOXM1 promotes cell proliferation in a variety of human malignancies including HCC [31–33], we investigated whether FOXM1 and KIF4A influence HCC progression. We stably expressed each protein in HepG2 cells (which have a relatively low endogenous expression of both factors) and silenced their expression in Huh7 and Hep3B HCC cells (which have relatively high endogenous levels of FOXM1 or KIF4A) using a lentiviral system. Cell proliferation was evaluated by the EdU, colony formation, cell growth, and CCK-8 assays. The overexpression or knockdown of FOXM1 and KIF4A in these cell lines was confirmed by western blotting (Additional file 3: Figure S2a, b). FOXM1 overexpression increased the percentage of EdU-positive HepG2 cells (Fig. 4a), reflecting a higher proportion of cells entering the DNA replication phase of the cell cycle. FOXM1 knockdown had the opposite effect on Huh7 cells (Fig. 4b). FOXM1 overexpression also increased HepG2 cell colony formation (Fig. 4e), growth (Fig. 4g), and viability (Fig. 4i) whereas the contrary was true upon FOXM1 knockdown in Huh7 cells (Fig. 4f, h, j). We also analyzed cell cycle distribution in FOXM1-depleted Hep3B cells by flow cytometry. Compared to control cells expressing a scrambled control shRNA (sh-Control), those expressing shRNA against FOXM1 (sh-FOXM1) were arrested in G1 phase (Fig. 4k). These findings provide additional evidence that FOXM1 promotes HCC cell proliferation.

The same assays were performed with KIF4A-overexpression or -deficient HCC cell lines. KIF4A overexpression increased cell proliferation rate, colony formation, growth rate, and viability of HepG2 cells (Fig. 4c, e, g, i), whereas the opposite effect was observed upon KIF4A knockdown in Huh7 cells (Fig. 4d, f, h, j). The latter was accompanied by inhibition of mitotic progression and cytokinesis, resulting in the accumulation of cells in G2/M phase and multinucleate cells (> 4 N cells) (Fig. 4l). These results demonstrate that FOXM1 and KIF4A play essential roles in promoting HCC cell proliferation.

### FOXM1 stimulates HCC cell proliferation in a KIF4A-dependent manner

**Fig. 5:**
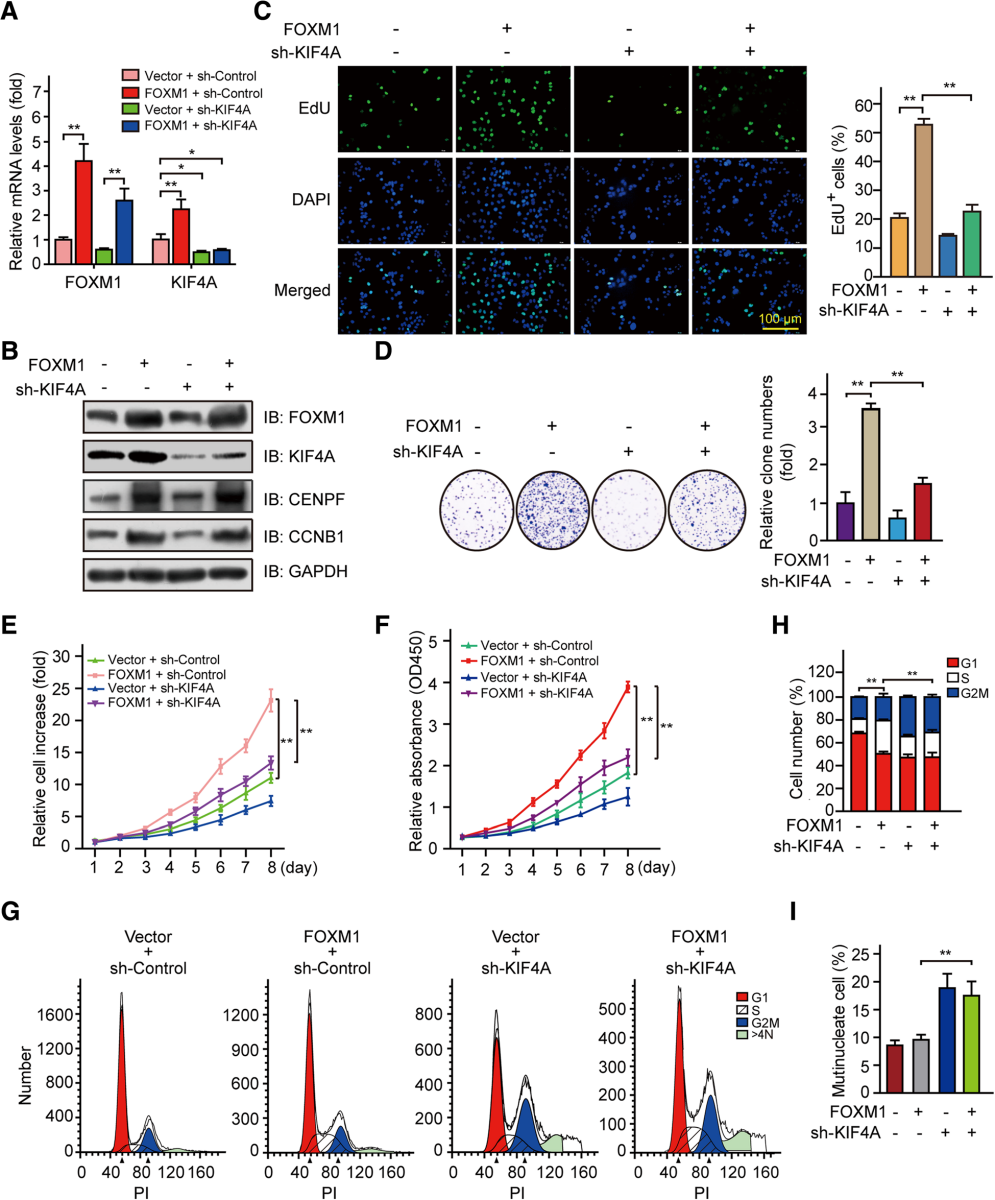
KIF4A is required for FOXM1-induced HCC cell proliferation in vitro. **a, b** HepG2 cells were infected with FOXM1 overexpression (FOXM1) and KIF4A knockdown (sh-KIF4A) lentiviral vectors and cells were harvested for quantitative real-time PCR (**a**) or western blotting (**b**). **c–f** Proliferation of HepG2 cells stably expressing FOXM1 + sh-KIF4A was evaluated with the EdU (**c**), colony formation (**d**), cell growth (**e**), and CCK-8 (**f**) assays. **g–i** HepG2 cells stably expressing FOXM1 + sh-KIF4A were subjected to cell cycle analysis (**g**); the fraction of cells in each phase (**h**) and number of multinucleate cells (**i**) were shown as a histogram. Data represent mean and SD of three independent experiments. *p < 0.05; **p < 0.01

Based on the above observations, we speculated that KIF4A functions as an effector of FOXM1 in the context of HCC progression. To test this hypothesis, we knocked down KIF4A in FOXM1-overexpressing HepG2 cells. The upregulation of KIF4A caused by FOXM1 overexpression was abrogated at both mRNA and protein levels (Fig. 5a, b). We analyzed cell proliferation rate with the EdU assay and found that it was increased in FOXM1 + sh-Control relative to Empty vector + sh-Control HepG2 cells, but this was abrogated by KIF4A knockdown (FOXM1 + sh-KIF4A) (Fig. 5c), suggesting that FOXM1 promotes HCC cell proliferation via KIF4A. The colony formation, cell growth, and CCK-8 assays yielded similar results (Fig. 5d-f). Finally, we examined whether KIF4A is also a downstream factor in FOXM1-induced cell cycle progression. KIF4A knockdown blocked HepG2 cell cycle progression, as evidenced by a higher fraction of cells in S phase caused by FOXM1 overexpression, G2/M phase arrest, and accumulation of multinucleate cells (Fig. 5g-i). These results demonstrate that the enhancement of HCC cell proliferation by FOXM1 depends on KIF4A.

### FOXM1–KIF4A axis modulates tumor growth in an HCC xenograft model

**Fig. 6:**
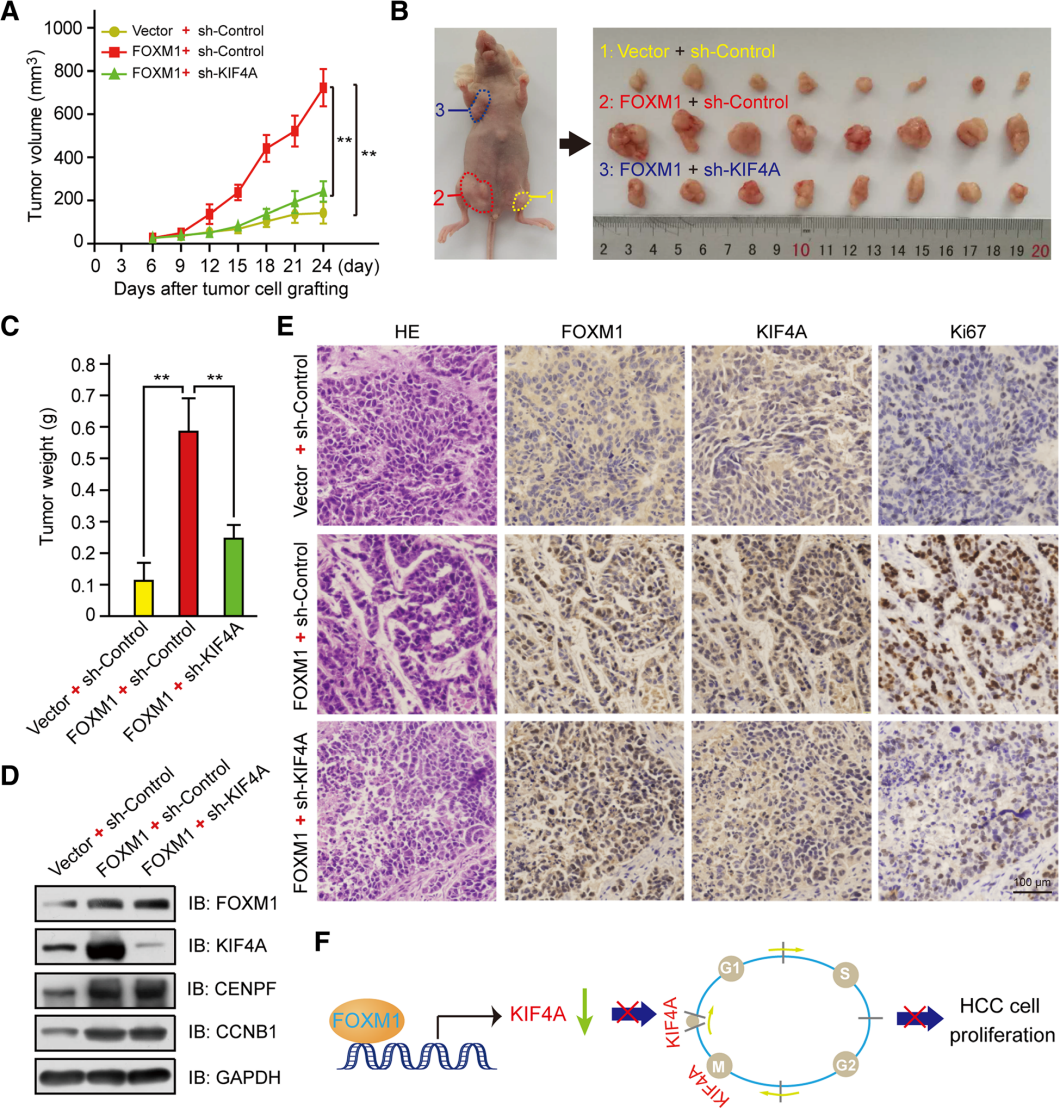
FOXM1–KIF4A axis promotes HCC progression in vivo. **a** Growth curves of xenograft tumors formed by indicated Huh7 cells in nude mice. Data represent mean ± SD (*n* = 8). **p < 0.01. **b** Image of xenograft tumors. **c** Weight of xenograft tumors. Data represent mean ± SD (n = 8). **p < 0.01. **d** Downregulation of KIF4A protein in Huh7 FOXM1 + sh-KIF4A compared to Huh7 FOXM1 + sh-Control xenografts, as confirmed by western blotting. **e** HE staining of tumor tissue samples from indicated groups and detection of FOXM1, KIF4A, and Ki67 protein levels by immunohistochemistry. **f** Model for FOXM1-KIF4A axis mediated HCC proliferation. FOXM1 transcriptionally activates KIF4A via directly binding to the promoter of KIF4A, promoting mitosis of cell cycle and proliferation of HCC cells. The inhibition of KIF4A is shown to suppress FOXM1-induced HCC proliferation and growth in this study

We investigated whether the above findings are applicable in vivo using an HCC mouse tumor xenograft model. We subcutaneously injected nude mice with Huh7 cells stably expressed with Vector + sh-Control, FOXM1 + sh-Control, or FOXM1 + sh-KIF4A. Compared to the control group, tumors in the FOXM1 + sh-Control group developed much more rapidly, but this effect was abrogated by KIF4A knockdown (FOXM1 + sh-KIF4A group) (Fig. 6a). Tumors were smaller in FOXM1 + sh-KIF4A than in FOXM1 + sh-Control mice (Fig. 6b, c), and FOXM1 + sh-KIF4A xenografts expressed lower levels of KIF4A as well as the proliferation marker Ki67 (Fig. 6e), as determined by immunohistochemistry and western blotting (Fig. 6d). These results indicate that the FOXM1–KIF4A axis promotes HCC progression and interfering the expression of KIF4A blocks FOXM1-mediated HCC cell proliferation (Fig. 6f).

## Discussion

FOXM1 is a vital regulator of many biological processes, and dysregulation of FOXM1 contributes to carcinogenesis and tumor progression [34]. A recent meta-analysis of 18,000 cases comprising 39 human malignancies showed that the FOXM1 regulatory network is a significant predictor of adverse clinical outcome [35]. However, the mechanisms of action of FOXM1 in human cancers remain unclear. Previous studies demonstrated that FOXM1b is essential for the development of HCC [36] and FOXM1 promotes EMT of HCC by regulating Snai1 [37]. In this study, we identified KIF4A as a direct target of FOXM1c in HCC and found that KIF4A expression was strongly correlated with that of FOXM1; moreover, elevated levels of FOXM1 and KIF4A in HCC were closely associated with poor prognosis. The pro-proliferative effect of FOXM1 was dependent on KIF4A and knockdown of KIF4A blocked the stimulation of FOXM1 on HCC cell proliferation.

*FOXM1* gene formats four distinct isoforms based on its two alternative splicing exons (Va and VIIa) [38]. FOXM1a, which contains both Va and VIIa, is poorly characterized due to its transcriptional inactivity. Though FOXM1d, which has only exon VIIa, is identified to promote cancer EMT and progression via interacting with ROCKs, it has no direct transcription regulating function due to its predominantly cytoplasmic location and disruption of the gene transactivation domain by the insertion of exon VIIa [2]. In contrast, FOXM1b, which contains neither of the two exons and FOXM1c, which has only Va, mainly function as transcriptional activators in the nucleus [39] and activate their target genes through different mechanisms [27]. FOXM1b regulates cell proliferation, EMT, angiogenesis, and metastasis by stimulating the expression of caveolin-1 [13], vascular endothelial growth factor [40], and matrix metalloproteinase (MMP)-2 [41]. FOXM1c differs from FOXM1b by the extra exon Va insertion that contains an extracellular signal-regulated kinase 1/2 target sequence that enables its activation by RAF/mitogen-activated protein kinase (MAPK)/MAP kinase signaling [42]. FOXM1c overexpression is associated with EMT and metastasis in pancreatic cancer [28] and with cell proliferation, anchorage-independent growth, migration, and invasion in ovarian cancer [43]. Additionally, FOXM1c promotes esophageal cancer metastasis through transcriptional regulation of interferon regulatory factor 1 expression [10]. In the present study, we showed that FOXM1b and FOXM1c but not FOXM1a promotes KIF4A expression, with FOXM1c exhibiting the main effect (Fig. 2a and b). Furthermore, we found that isoform FOXM1c directly regulates the expression of KIF4A by ChIP and the luciferase reporter assay (Fig. 3). Our data together with the previous studies further confirm the pivotal role of FOXM1c in cancer cell progression. FOXM1d is reported to locate in the cytoplasmic and does not directly control transcription, but it can interact with ROCKs and activate the activity of ROCKs to promote colorectal cancer EMT and metastasis [2]. In our study, we also found FOXM1d, to a small extent, induced KIF4A expression (Fig. 2a and b); however, the molecular mechanism needs to be further elucidated.

FOXM1 is involved in many solid tumors [44], and it regulates a wide range of proliferative signals in a cell cycle-dependent manner [45]. FOXM1 expression is relatively low in quiescent cells but elevated in most tumors including liver cancer [33, 46–48]. As a transcription factor, FOXM1 has many target genes including KIF20A, CENPF, CCNB1, MYC, NIMA-related kinase 2, MMP2, MMP9, and S-phase kinase-associated protein 2 that are implicated in cancer initiation, progression, or drug resistance [3, 9, 49]. The present study advances our understanding of the mechanism by which FOXM1 regulates HCC progression in several ways. Firstly, our immunohistochemistry analyses showed that FOXM1 and KIF4A protein expression was upregulated in clinical HCC tissue specimens and that this was positively associated with poor prognosis of HCC patients. Secondly, this is the first report to demonstrate that KIF4A is a direct target of FOXM1. These findings provide clinical and molecular evidence that FOXM1 regulates the expression of KIF4A to promote HCC progression.

KIF4A as a member of the kinesin family plays essential roles in DNA repair, DNA replication, spindle organization and cytokinesis [17]. KIF4A is aberrantly expressed in a variety of cancers, and KIF4A is overexpressed in most tumors but also low-expressed in a few tumors [17], suggesting its distinct functions and mechanisms for different tumors. In the present study, we demonstrate that KIF4A is a downstream target of FOXM1 and directly regulated by FOXM1 through interacting with the binding site of BS3 (5′-AGATGGAGT-3′) in the KIF4A promoter. Moreover, we found that KIF4A is required for the FOXM1-mediated promotion of HCC cell proliferation and silencing of KIF4A reversed the FOXM1 promotion of HCC cell proliferation. Particularly, knockdown of KIF4A blocked the cell cycle progression induced by FOXM1 and produced multinucleate cells of HCC. The reason for cell cycle delay and increase of multinucleate cells maybe because depletion of KIF4A caused mitotic spindle defects, anaphase bridges and defective cytokinesis [50, 51]. Our results provide a new insight that FOXM1 regulates cell cycle based on the regulation of KIF4A.

Owing to the well-known roles in tumor progression, FOXM1 is a potential therapeutic target in many human cancers [52]. For instance, the natural inhibitor agent thiostrepton blocks the transcriptional activity of FOXM1 by preventing its binding to target sites in target gene promoters [53], leading to the suppression of tumor growth [54]. The thiazole compound siomycin A also inhibits FOXM1 transcriptional activity [55]. However, these identified drugs are also associated with severe side effects or drug resistance [56]. Our results showed that knocking down KIF4A expression suppresses FOXM1 mediated HCC cell proliferation and tumor growth, suggesting that FOXM1–KIF4A axis may be a potential therapeutic target for HCC treatment. Even though, many questions remain concerning the function of the FOXM1-KIF4A axis. For example, whether the FOXM1-KIF4A signaling axis has some other mechanisms to promote HCC progression or does this axis work on other tumors? Do other specific factors regulate the activity of KIF4A? Answering these questions will help us understand the mechanisms of transcription control and tumorigenesis of HCC and even other cancers.

## Conclusions

In summary, to our knowledge, we showed for the first time that KIF4A is a novel direct downstream target of FOXM1, which is required for FOXM1-mediated HCC proliferation. Our findings suggest that therapeutic strategies targeting the FOXM1–KIF4A axis can be useful for the treatment of HCC and possibly other cancers that are characterized by FOXM1 overexpression.

## Additional files

Additional file 1: **Table S1.** HCC datasets downloaded from NCBI GEO. **Table S2.** The shRNAs used for specific genes knockdown. **Table S3.** Primers used for the construction of luciferase reporter constructs. **Table S4.** Primers used for real-time PCR amplification. **Table S5.** Primers used for ChIP assay. (DOC 49 kb)

Additional file 2: **Figure S1.** FOXM1 and KIF4A expression levels are positively correlated in HCC tissue. a–c mRNA expression of FOXM1 (a), KIF4A (b), and CENPF and KIF20A (c), in human HCC and non-tumor tissues based on data from TCGA. d, e KIF4A, and FOXM1 expression is positively correlated (d) and CENPF and KIF20A expression is positively associated with that of FOXM1 (e) according to data from the cBioPortal for Cancer Genomic database. f Correlation between FOXM1 and KIF4A expression and pathological grade of tumors. Three serial sections of HCC tissue were labeled with anti-FOXM1 and -KIF4A antibodies. Representative images from three cases with different degrees of histological differentiation (well to poorly differentiated) are shown. g Expression scores of FOXM1 and KIF4A are shown as box plots. The number of samples for each grade is shown below the group. Data were analyzed with the Kruskal–Wallis H test. h, i Overall survival rate associated with FOXM1 (h) and KIF4A (i) based on records in TCGA. Data in Kaplan-Meier curves were analyzed with the log-rank test. (TIF 5320 kb)

Additional file 3: **Figure S2.** Effect confirmation of the lentivirus infected HCC cell lines. a HepG2 cells infected with lentivirus of FOXM1 or KIF4A overexpression. b Huh7 cells infected with FOXM1 or KIF4A knockdown lentivirus and Hep3B cells infected with FOXM1 knockdown lentivirus. (TIF 802 kb)

## Abbreviations

BS: Binding site
CCK-8: Cell Counting Kit-8
CCNB1: Cyclin B1
CENPF: Centromere protein F
ChIP: Chromatin immunoprecipitation
EdU: 5-ethynyl-2′-deoxyuridine
EMT: Epithelial-mesenchymal transition
GAPDH: Glyceraldehyde 3-phosphate dehydrogenase
GEO: Gene Expression Omnibus
HBV: Hepatitis B virus
HCC: Hepatocellular carcinoma
HE: Hematoxylin-eosin
PBS: Phosphate-buffered saline
RIPA: Radioimmunoprecipitation
shRNA: short hairpin RNA
TCGA: The Cancer Genome Atlas
TNM: Tumor–node–metastasis
