# Supplementary material for: FOXM1 promotes hepatocellular carcinoma progression by regulating KIF4A expression
Source: J Exp Clin Cancer Res. 2019 May 9;38:188. doi: 10.1186/s13046-019-1202-3 (PMC6507024; doi:10.1186/s13046-019-1202-3)
Supplement: Supplementary file 1 — Table S1. HCC datasets downloaded from NCBI GEO. Table S2. The shRNAs used for specific genes knockdown. Table S3. Primers used for the construction of luciferase reporter constructs. Table S4. Primers used for real-time PCR amplification. Table S5. Primers used for ChIP assay. (DOC 49 kb) [file 13046_2019_1202_MOESM1_ESM.doc]

**Additional file 1: Table S**1: HCC datasets downloaded from NCBI GEO

| GEO ID | Platforms | Total | Non-tumor | Tumor |
| --- | --- | --- | --- | --- |
| GSE6764 | HG-U133_Plus_2 | 58 | 23 | 35 |
| GSE14520_1 | HG-U133A_2 | 395 | 189 | 206 |
| GSE14520_2 | HT_HG-U133A | 43 | 21 | 22 |
| GSE41804 | HG-U133_Plus_2 | 40 | 20 | 20 |
| GSE45267 | HG-U133_Plus_2 | 86 | 41 | 45 |
| GSE60502 | HG-U133A | 28 | 15 | 13 |
| Total |  | 650 | 309 | 341 |

**Table S2: The shRNAs used for specific genes knockdown**

| ID | Target sequences (5’-3’) | Start | Remark |
| --- | --- | --- | --- |
| shRNA-FOXM1 (1#) | 5’-GCCCAACAGGAGTCTAATCAA-3’ | 109 |  |
| shRNA-FOXM1(2#) | 5’-TGATACAATTCGCCATCAACA-3’ | 731 |  |
| shRNA-FOXM1 (3#) | 5’-TGTCTCGGAAATGCTTGTGAT-3’ | 1692 | For Lentivirus shRNA System |
| shRNA-KIF4A (1#) | 5’-GAGCATGAGGATGGTGATGGT-3’ | 3303 | For Lentivirus shRNA System |
| shRNA-KIF4A(2#) | 5’-CATGAGGATGGTGATGGTGAT-3’ | 3306 |  |
| shRNA-KIF4A (3#) | 5’-GATGGTGATGGTGATGATGAT-3’ | 3312 |  |

**Table S3**: Primers used for construction of luciferase reporter constructs

| Promoters | Range | Forward primer (5’ to 3’) | Reverse primer (5’ to 3’) |
| --- | --- | --- | --- |
| pKIF4A-full | -1810 ~ -764 | 5’-CCGCTCGAGCACAAGAACAACTAGTAGC  ATCATCGTAC-3’ | 5’-CCCAAGCTTCTCCTCATGAGGTGGGAA  CCATCTG-3’ |
| pKIF4A-BS2M | Mutation | 5’-CAACGAATTGTTGTAGTCCGGGTGGTG-3’ | 5’-ACCCAGTTTCTGCCCCGAACCATC-3’ |
| pKIF4A-BS3M | Mutation | 5’-TTTTTTGAGATGTCGTTTTGCTCTTGTTGCC  CAGG-3’ | 5’-AAAAAAAAAAGGTGAAGAAATGGGGAG  C-3’ |
| pKIF4A-BS4M | Mutation | 5’-AGATTTCCTCTCCGTTTCGCCTTCTAACCC  TGC-3’ | 5’-TAGCACTTGTAGCCAGAGGACCCAG-3’ |

**Table S4**: Primers used for real-time PCR amplification

| Genes | Forward primer (5’ to 3’) | Reverse primer (5’ to 3’) |
| --- | --- | --- |
| FOXM1 | 5’-ACCGCTACTTGACATTGGAC-3’ | 5’-GGGAGTTCGGTTTTGATGGTC-3’ |
| KIF4A | 5’-GGGATGACGAGGAATGGAAG-3’ | 5’-TCACAGCAACAGTCCACAC-3’ |
| GAPDH | 5’-CACCAGGGCTGCTTTTAACTCTG-3’ | 5’-GATTTTGGAGGGATCTCGCTCCTG-3’ |

**Table S5: Primers used for ChIP assay**

| Gene | Predictive BS (5’ to 3’) | Forward primer (5’ to 3’) | Reverse primer (5’ to 3’) |
| --- | --- | --- | --- |
| KIF4A | BS1: 5’-AGATTCTTT-3’ | 5’-GGAAGGGGTGAAAACAGCGTCTC-3’ | 5’-CCTGAAAGGAGGATGATGATATCTAGG-3’ |
|  | BS2: 5’-ACTCATTGT-3’ | 5’-CCAATTTGTGGTATGGATGAATCTAGC-3’ | 5’-TGCCCCAGTGCTATCCATCTACTC-3’ |
|  | BS3: 5’-AGATGGAGT-3’ | 5’-GATGGATAGCACTGGGGCAGC-3’ | 5’-GCACATGCCTGTAATCCCAGC-3’ |
|  | BS4: 5’-AGATTCACT-3’ | 5’-CCAGAATTAACCGGTGTGAACAG-3’ | 5’-TCTTGCCTGCCTATGAGAATCC-3’ |

BS: Binding Sequences.
