# Supplementary figures and images for: FOXM1 promotes hepatocellular carcinoma progression by regulating KIF4A expression
Source: J Exp Clin Cancer Res. 2019 May 9;38:188. doi: 10.1186/s13046-019-1202-3 (PMC6507024; doi:10.1186/s13046-019-1202-3)

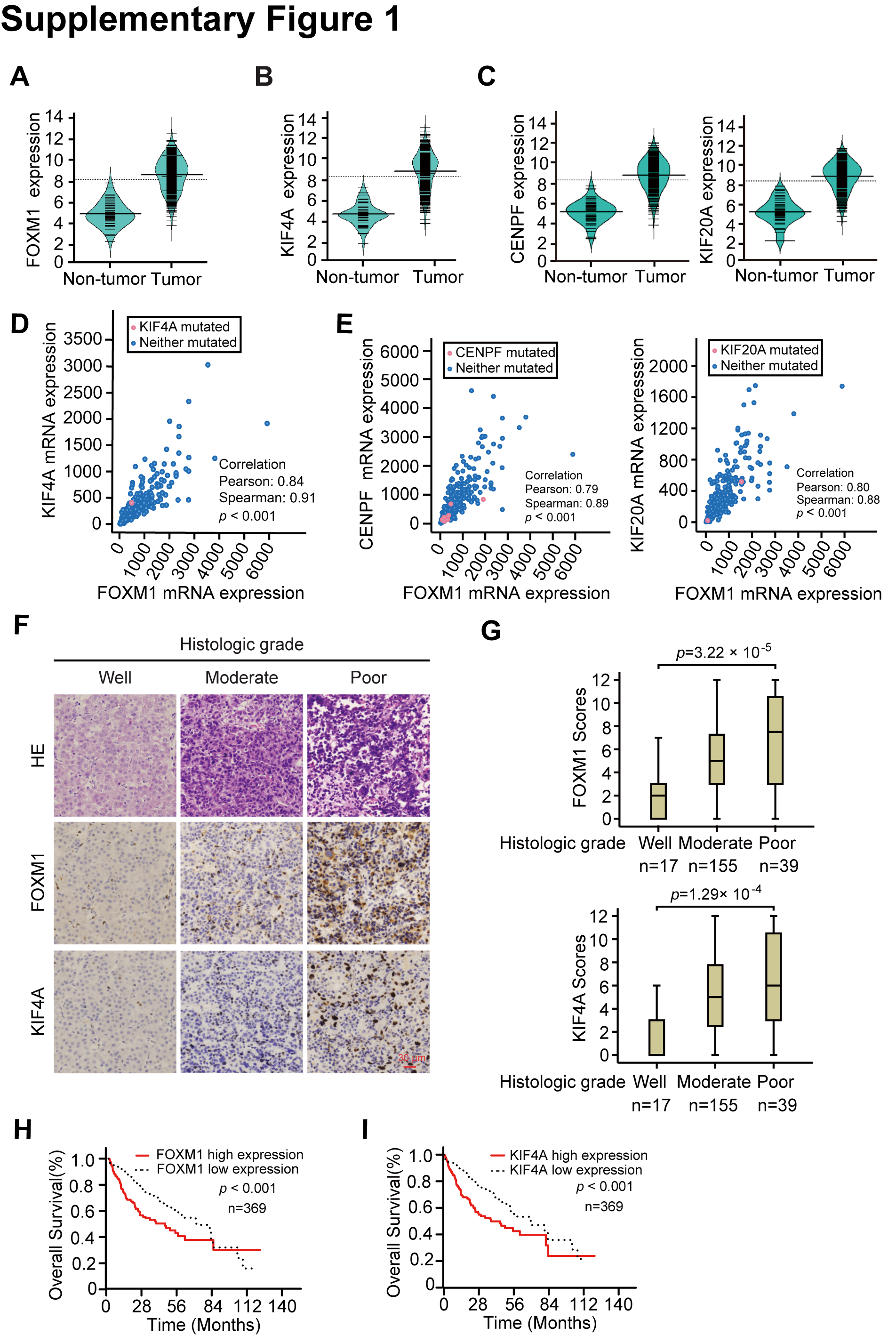

Supplement: Supplementary file 2 — Figure S1. FOXM1 and KIF4A expression levels are positively correlated in HCC tissue. a–c mRNA expression of FOXM1 (a), KIF4A (b), and CENPF and KIF20A (c), in human HCC and non-tumor tissues based on data from TCGA. d, e KIF4A, and FOXM1 expression is positively correlated (d) and CENPF and KIF20A expression is positively associated with that of FOXM1 (e) according to data from the cBioPortal for Cancer Genomic database. f Correlation between FOXM1 and KIF4A expression and pathological grade of tumors. Three serial sections of HCC tissue were labeled with anti-FOXM1 and -KIF4A antibodies. Representative images from three cases with different degrees of histological differentiation (well to poorly differentiated) are shown. g Expression scores of FOXM1 and KIF4A are shown as box plots. The number of samples for each grade is shown below the group. Data were analyzed with the Kruskal–Wallis H test. h, i Overall survival rate associated with FOXM1 (h) and KIF4A (i) based on records in TCGA. Data in Kaplan-Meier curves were analyzed with the log-rank test. (TIF 5320 kb) [file 13046_2019_1202_MOESM2_ESM.tif]

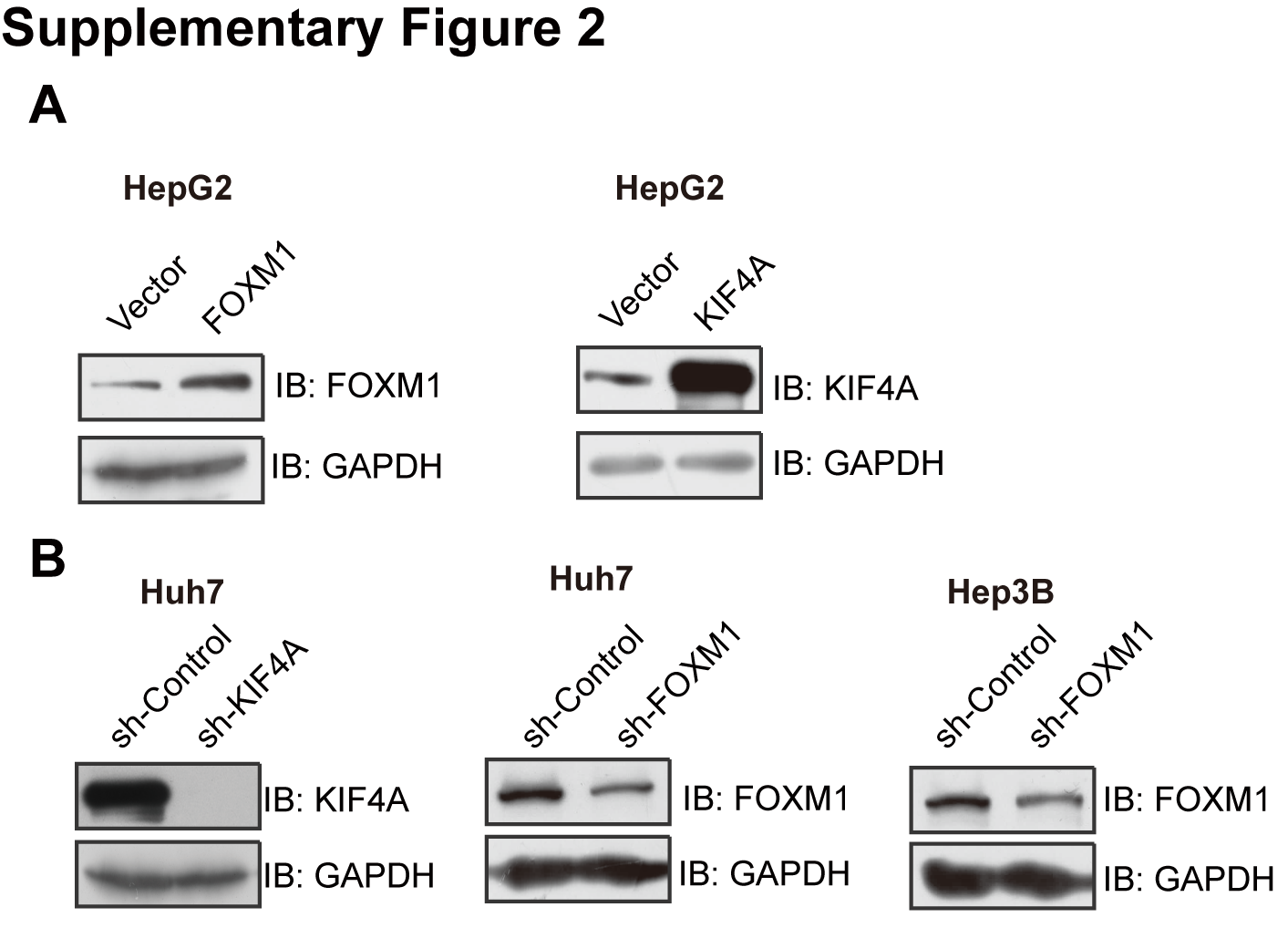

Supplement: Supplementary file 3 — Figure S2. Effect confirmation of the lentivirus infected HCC cell lines. a HepG2 cells infected with lentivirus of FOXM1 or KIF4A overexpression. b Huh7 cells infected with FOXM1 or KIF4A knockdown lentivirus and Hep3B cells infected with FOXM1 knockdown lentivirus. (TIF 802 kb) [file 13046_2019_1202_MOESM3_ESM.tif]
